# Succinate Prevents Mice Obesity by Enhancing Brown Adipocyte Thermogenesis via the SDH-METTL3-HIF1A Pathway

**DOI:** 10.3390/ijms27125348

**Published:** 2026-06-13

**Authors:** Yaojun Luo, Zimeng Xin, Youhua Liu, Ruiti Ren, Xinxia Wang

**Affiliations:** 1 National Engineering Research Center of Green Feeds and Healthy Livestock Industry, Zhejiang University, Hangzhou 310058, China; yaojunluo@zju.edu.cn (Y.L.); meng1217180507@163.com (Z.X.);; 2 College of Animal Sciences, Zhejiang University, No. 866 Yuhangtang Road, Hangzhou 310058, China; 3 Key Laboratory of Molecular Animal Nutrition, Zhejiang University, Ministry of Education, Hangzhou 310058, China; 4 Key Laboratory of Animal Nutrition and Feed Science (Eastern China), Ministry of Agriculture and Rural Affairs, Hangzhou 310058, China; 5 Zhejiang Key Laboratory of Nutrition and Breeding for High-Quality Animal Products, Hangzhou 310058, China

**Keywords:** succinate, obesity, brown adipocytes, m^6^A

## Abstract

Succinate, a tricarboxylic acid (TCA) cycle intermediate, is the essential signal molecule that links metabolic signals and inflammation. Dietary succinate supplementation has been reported to prevent obesity induced by a high-fat diet (HFD). However, the underlying mechanism remains elusive. Here, we found that dietary succinate elevated the serum succinate levels. Meanwhile, we found succinate increased methyltransferaselike 3 (METTL3) protein expression in brown adipocytes, thereby elevating N^6^-methyladenosine (m^6^A) levels in Hypoxia-inducible factor1-alpha (*Hif1a*) mRNA. *Hif1a* mRNA is recognized by the m^6^A-binding protein YTH domain-containing family protein 1 (YTHDF1), facilitating HIF1A protein expression. HIF1A activates the transcription of thermogenic genes, ultimately increasing brown adipose energy expenditure. Together, our research provided new insights into the effect of succinate on m^6^A modification in brown adipose tissue thermogenesis.

## 1. Introduction

Obesity, characterized by increased adipose tissue mass, is linked with metabolic syndrome, which represents a significant health concern in the world [1,2]. It is urgent to develop new therapeutic strategies to ameliorate obesity. Essentially, obesity ensues from an imbalance between energy intake and expenditure. Reducing energy intake or enhancing energy expenditure is a reasonable strategy to combat obesity. Brown adipose tissue (BAT) is an organ that can dissipate energy in the form of heat. Previous studies have shown that BAT is a promising organ for combating obesity [3,4,5]. However, brown adipose converts chemical energy to heat, which requires activation by external stimuli [6]. Meanwhile, our current understanding of dietary molecules involved in BAT thermogenesis is limited.

Succinate, a TCA cycle intermediate, plays crucial roles in metabolism [7,8], inflammation signal [9,10], and epigenetics [11,12]. Recent discoveries suggest that succinate can be taken up into brown adipocytes and be oxidized by succinate dehydrogenase (SDHB) to generate mitochondrial Reactive oxygen species (ROS), which activate thermogenesis [6]. However, several surveys have provided little evidence that succinate supplementation in the diet or drinking water could impact circulating succinate levels [13]. In addition, as a microbial metabolite, succinate could be yielded by *Bacteroides* spp. *Prevotella* spp., *Firmicutes* spp., and other bacteria that inhabit the gut [14]. It remains unclear whether dietary succinate can enter the circulatory system and ultimately be taken up into brown adipocytes, triggering thermogenesis. Meanwhile, succinate accumulation in cells may increase lysine succinylation, which regulates energy homeostasis. However, the underlying mechanisms by which succinate triggers brown adipose tissue thermogenesis via epigenetic modification remain poorly understood.

m^6^A, the most abundant modification on mRNAs in eukaryotes, plays essential roles in obesity and energy homeostasis [15,16,17,18]. Natural compounds have been proven to influence m^6^A methylase or demethylase activity or expression, which ultimately changes m^6^A levels. Notably, succinate has been reported to suppress (Fat mass and obesity-associated) FTO expression, thereby increasing m^6^A methylation levels and promoting Messenchymal stem cell (MSC) osteogenesis [19]. METTL3, the m^6^A methylase, constitutes a methyltransferase complex with methyltransferase 14 (METTL14), which plays the key role in catalyzing m^6^A formation [20,21]. Previous studies have shown that *Mettl3* is essential for brown adipose development and thermogenesis [16,17]. However, there is little evidence that succinate promotes brown adipose tissue thermogenesis via m^6^A modification. And it is also unknown whether succinate changes the expression of the METTL3 protein.

Our results showed that dietary succinate elevated circulating succinate levels, thereby inhibiting HFD-induced obesity and metabolic disorders through promoting brown adipose thermogenesis. Mechanistically, we revealed that succinate dehydrogenase is upstream of *Mettl3* and succinate enhances *Mettl3* expression. The augmented METTL3 increases m^6^A modification of *Hif1a* mRNA, subsequently HIF1A binds the thermogenic gene to trigger brown adipocyte thermogenesis in a YTH domain-containing family protein 1 (*Ythdf1*)-dependent manner. Together, we found that dietary succinate alleviated HFD-induced obesity by promoting brown adipose thermogenesis via RNA m^6^A modification.

## 2. Results

### 2.1. Succinate Prevents HFD-Induced Obesity in Mice

**Figure 1:**
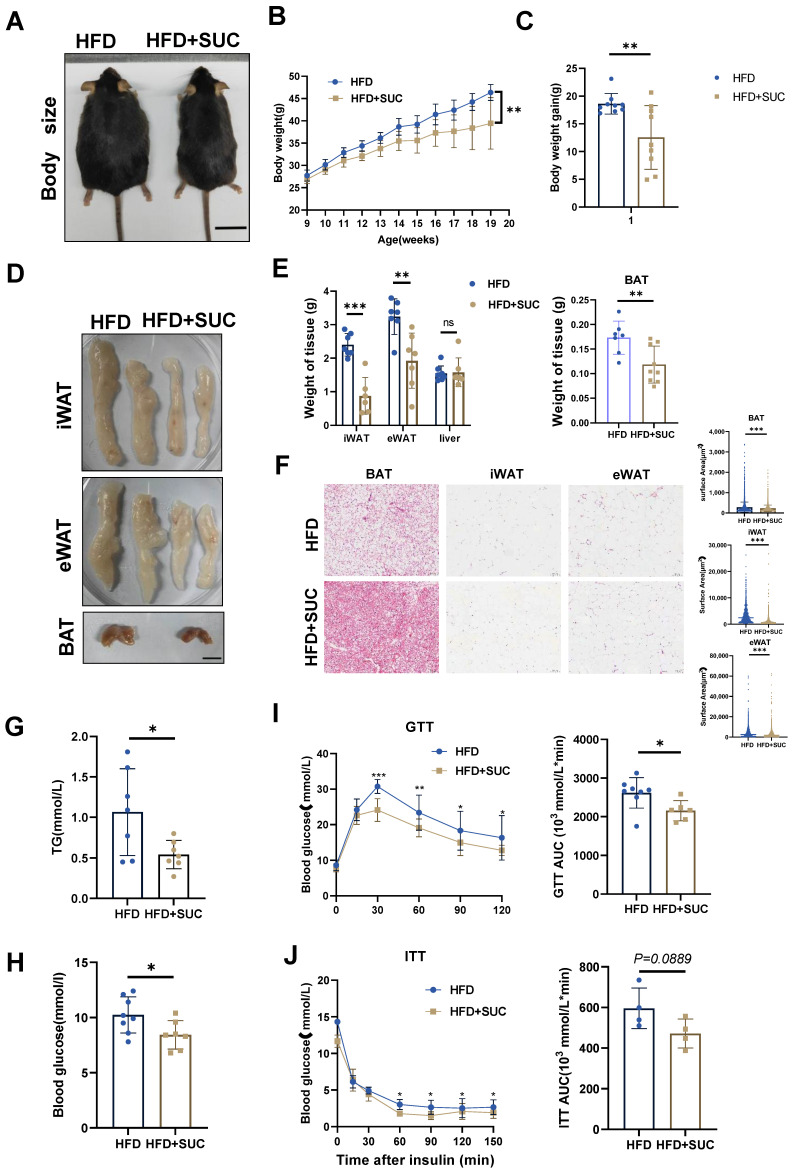
Succinate prevents HFD-induced mice obesity (**A**) Representative image of HFD-fed and HFD + SUC-fed mice (*n* = 9). Scale bar, 2cm. (**B**) Body weight trajectories of the mice from 9 weeks of age (*n* = 9). (**C**) body weight gain of mice at termination of study (*n* = 9). (**D**) Representative appearance of eWAT, iWAT, and BAT in mice (*n* = 7). Scale bar, 1 cm. (**E**) Weight for iWAT, eWAT, and BAT of HFD group and HFD + SUC group mice. (**F**) Representative H&E staining of iWAT, eWAT, and BAT from HFD and HFD + SUC group mice, adipocyte surface area in BAT, iWAT, and eWAT, of HFD group and HFD + SUC group mice. Scale bar, 200 μm. (**G**) Serum triglyceride levels in two groups of mice (*n* = 7). (**H**) Blood glucose levels in two groups of mice (*n* = 8). (**I**) Blood glucose levels of mice after intraperitoneal injection of insulin for Glucose tolerance tests (GTT). The area under the curve (AUC) was calculated based on GTT results (**J**) and blood glucose levels in mice after intraperitoneal injection of insulin for insulin tolerance tests (ITT). The area under the curve (AUC) was calculated based on ITT results. The data were presented as the mean ± SD by Student’s *t*-test (**C**,**E**–**J**). Two-way ANOVA was followed by Bonferroni’s test, which was used for repeated measurement of two groups (**B**,**I**,**J**). * *p* < 0.05, ** *p* < 0.01, *** *p* < 0.001.

To explore the effects of succinate on obesity, we fed the mice a high-fat diet (HFD) or a high-fat diet supplemented with succinate (HFD + SUC) for 10 weeks. As expected, we found that succinate-supplemented (HFD + SUC) group mice showed leaner body morphology than HFD-fed mice (Figure 1A). Meanwhile, succinate supplementation attenuated HFD-induced body weight gain (Figure 1B,C), while the food intake and water intake in mice were increased by succinate supplementation (Figure S1A,B). Consistently, the size and mass of mice inguinal WAT (iWAT), epididymal WAT (eWAT), and brown adipose tissue (BAT) were smaller and lower by succinate treatment compared with the high-fat diet condition (Figure 1D,E). Meanwhile, hematoxylin-eosin (H&E) staining revealed that the adipocyte size of eWAT, iWAT, and BAT was markedly decreased by succinate supplementation (Figure 1F). Moreover, serum triglyceride (TG) levels in mice under the treatment of succinate were significantly decreased (Figure 1G). And we measured serum glucose levels in HFD and HFD + SUC-fed mice; the results showed that succinate supplementation in HFD reduced fasting blood glucose levels related to normal HFD conditions (Figure 1H). Furthermore, we examined the effect of succinate on glucose tolerance and insulin sensitivity. As expected, HFD + SUC group mice showed enhanced glucose disposal ability and insulin sensitivity (Figure 1I,J), suggesting succinate preserves glucose homeostasis. Taken together, these results demonstrate that succinate prevents HFD-induced obesity and metabolic dysfunctions.

### 2.2. Succinate Enhances Mice Energy Expenditure by Promoting Brown Adipocyte Thermogenesis

**Figure 2:**
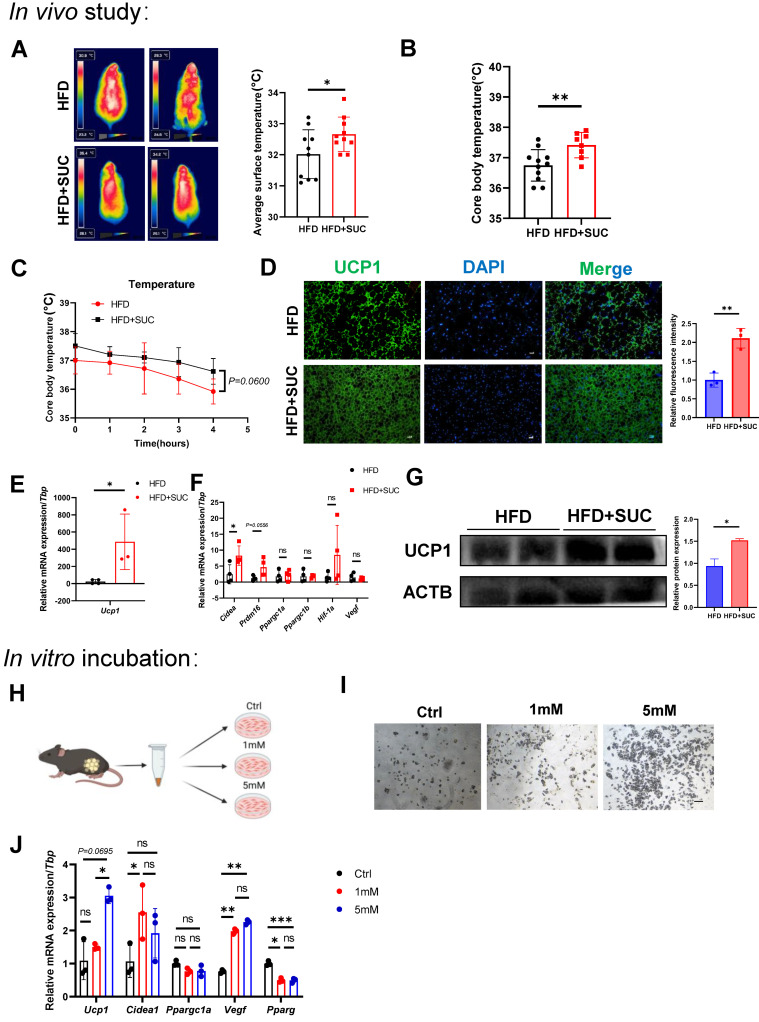
Succinate promotes brown adipocyte thermogenesis. (**A**) Thermal images and surface temperature of HFD and HFD + SUC fed mice (*n* = 10) (**B**) The rectal temperature of HFD and HFD + SUC fed mice (*n* = 10) (**C**) The rectal temperature of HFD and HFD + SUC fed mice during acute cold exposure (*n* = 6). (**D**) Representative immunofluorescence images of UCP1 protein in HFD and HFD + SUC fed mice’s brown adipose tissue. Scale bars, 100 µm (**E**) mRNA expression of *Ucp1* in brown adipose tissue of HFD and HFD + SUC fed mice. (**F**) Thermogenic gene expression in brown adipose tissue of HFD and HFD + SUC fed mice (*n* = 4). (**G**) Western blot analysis of UCP1 protein in HFD and HFD + SUC fed mice’s brown adipose tissue. (**H**) Schematic of cellular experiments in vitro. The primary stromal vascular fraction (SVF) cells were isolated from the BAT of male C57BL/6J mice, induced to differentiate, and treated with 0, 1, or 5 mM of succinate during the differentiation process. (**I**) Representative bright field images of primary brown preadipocytes after treating with 0, 1, and 5 mM succinate for 5 days. Scale bar, 50 μm. (**J**) mRNA expression of *Ucp1*, *Cidea*, *Ppargc1a*, *Vegf*, and *Pparg* in primary brown preadipocytes after treating with 0, 1, and 5 mM succinate. The data were presented as the mean ± SD by Student’s *t*-test (**A**,**B**,**E**–**G**,**I**). Two-way ANOVA was followed by Bonferroni’s test, which was used for repeated measurement of two groups (**C**). * *p* < 0.05, ** *p* < 0.01, *** *p* < 0.001.

Recently, several studies have shown that succinate can activate brown adipose tissue thermogenesis [6,22]. And we speculated that succinate promoted whole-body energy expenditure in diet-induced obesity. To validate that, we utilized an infrared imager to detect the surface body temperature. The imaging showed that succinate markedly elevated the surface temperature of mice fed with HFD (Figure 2A). Consistently, HFD + SUC group mice displayed higher core body temperature (Figure 2B). Compared to HFD-fed mice, HFD + SUC group mice exhibited higher temperature when exposed at 4 °C (Figure 2C). Immunofluorescence analysis revealed that succinate enhanced uncoupling Protein 1 (UCP1) expression in the BAT of HFD-fed mice (Figure 2D). Meanwhile, succinate supplementation increased the gene expression of mice BAT involved in thermogenesis, including *Ucp1* and *Cidea*. (Figure 2E,F). There were no changes in the thermogenesis-related gene, including *Ppargc1a*, *Ppargc1b*, and *Hif1a*, between the HFD and HFD + SUC group mice’s brown adipose tissue (Figure 2F). Consistently, the protein abundance UCP1 was also elevated in the BAT of HFD +SUC fed mice (Figure 2G). Previous studies have shown that endogenous succinate production is significantly higher than intestinal succinate absorption in normal physiological conditions [13]. We aim to determine whether dietary succinate enters the circulating system and promotes brown adipose thermogenesis. Therefore, we detected the concentration of succinate in serum. The results show that succinate supplementation markedly increases the serum succinate levels (Figure S2A). To further confirm that succinate prevents HFD-induced obesity through thermogenesis rather than inhibiting white adipose tissue hypertrophy. We treated primary iWAT preadipocytes with succinate; however, succinate promoted iWAT preadipocytes’ adipogenesis (Figure S2D). Meanwhile, we treated eWAT preadipocytes with succinate in three-dimensional (3D) culture conditions, and the results suggested that succinate promotes eWAT preadipocytes adipogenesis (Figure S2E). To investigate the role of succinate in brown adipogenesis, we isolated brown preadipocytes from mice and treated them with different doses of succinate during brown adipogenesis (Figure 2H). The phase image showed that succinate promoted the formation of more multilocular brown adipocytes in a dose-dependent manner in vitro (Figure 2I). Consistent with the result, succinate supplementation increased mRNA levels of thermogenic genes, including *Ucp1*, *Cidea1*, and *Vegf* in vitro (Figure 2J).

### 2.3. Succinate Promotes Brown Adipocyte Thermogenesis by Enhancing METTL3 Expression

**Figure 3:**
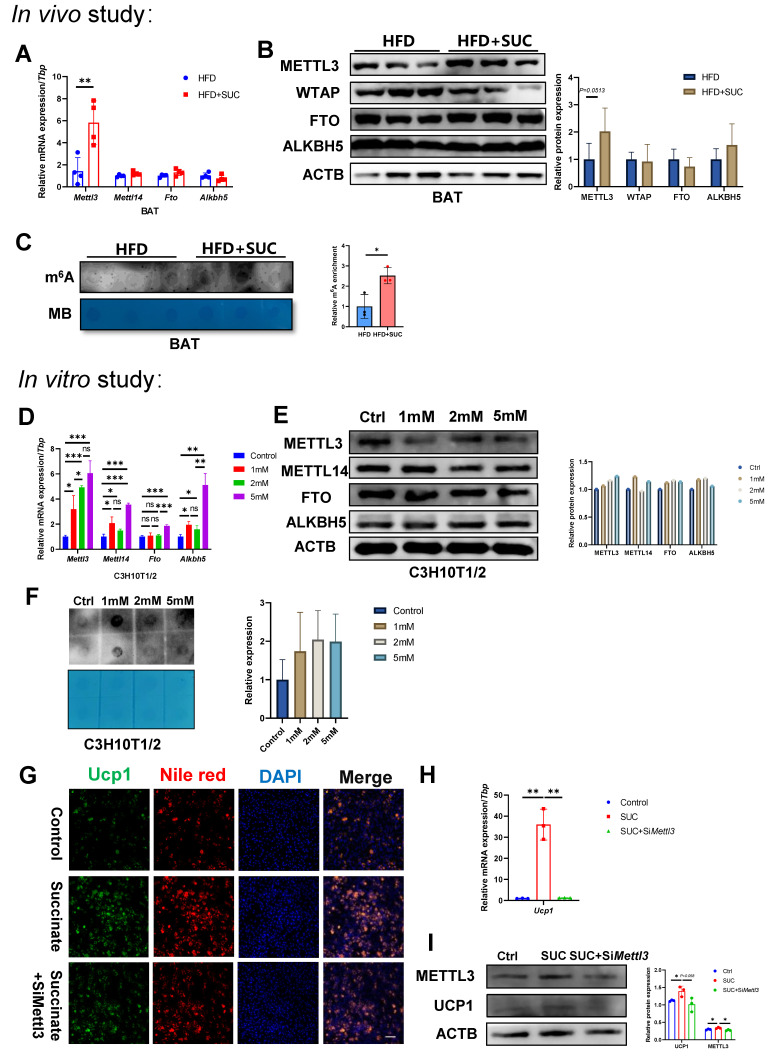
Succinate promotes brown adipocyte thermogenesis by enhancing METTL3 expression. (**A**) qPCR analysis of *Mettl3*, *Mettl14*, *Fto*, and *Alkbh5* in BAT from HFD and HFD + SUC fed mice (*n* = 4). (**B**) Western blot analysis of METTL3, WTAP, FTO, and ALKBH5 proteins in BAT from HFD and HFD + SUC fed mice. (**C**) Dot blot analysis for total m^6^A level in BAT of HFD group and HFD + SUC group mice (*n* = 3). (**D**) qPCR analysis of *Mettl3*, *Mettl14*, *Fto*, and *Alkbh5* in C3H10T1/2 cells treated with 0, 1, 2 and 5 mM succinate. (**E**) Western blot analysis of METTL3, METTL14, FTO, and ALKBH5 proteins in C3H10T1/2 cells treated with 0, 1, 2 and 5 mM succinate. (**F**) Dot blot analysis for total m^6^A level in C3H10T1/2 cells treated with 0, 1, 2 and 5 mM succinate. (**G**) Immunofluorescence of C3H10T1/2 cells in Ctrl, SUC-treated, and *Mettl3*-silenced groups. Scale bar, 50 μm. (**H**) qPCR analysis of *Ucp1* mRNA level in control, succinate-treated and *Mettl3*-silenced groups C3H10T1/2 cells. (**I**) Westernblot analysis of UCP1 protein expression in control, succinate-treated, and *Mettl3*-silenced groups C3H10T1/2 cells. The data were presented as the mean ± SD by Student’s *t*-test (**A**–**C**,**E**,**F**,**H**). * *p* < 0.05, ** *p* < 0.01, *** *p* < 0.001.

Because previous studies have shown that *Mettl3* is essential for brown adipose development and thermogenesis [16,17]. We hypothesized that succinate supplementation might influence m^6^A levels via m^6^A writers or erasers. To determine whether succinate promotes thermogenesis by affecting m^6^A writers or erasers, qPCR was performed on BAT from mice fed with HFD or HFD + SUC. The results showed that succinate increased *Mettl3* mRNA level in BAT (Figure 3A). Meanwhile, we detected the protein expressions of FTO, ALKBH5, WTAP, and METTL3. As expected, succinate also elevated METTL3 protein expression in BAT (Figure 3B). Consistently, we showed that *Mettl3* mRNA and protein expression were also higher in succinate-treated cells compared with the control group (Figure 3D,E). As a crucial m^6^A methyltransferase, augmented METTL3 usually elevates the m^6^A level. Therefore, we performed dot-blot analysis of RNA from the brown tissue of two groups of mice and succinate-treated C3H10T1/2 cells. The results showed that succinate increased m^6^A levels in BAT and C3H10T1/2 cells (Figure 3C,F). To further confirm that succinate promotes brown adipocyte thermogenesis via METTL3 protein, we conducted loss-of-function studies in C3H10T1/2 cells with control or *Mettl3* siRNA. The results showed that the absence of METTL3 could inhibit browning of C3H10T1/2 cells facilitated by succinate and UCP1 expression of C3H10T1/2 cells (Figure 3G–I). On the contrary, elevated METTL3 protein markedly facilitates C3H10T1/2 cells thermogenesis and UCP1 mRNA and protein expression (Figure S3B–D). Overall, these results suggested that succinate accelerated brown adipocyte thermogenesis via METTL3 and influenced m^6^A levels of brown adipocytes in vivo or in vitro.

### 2.4. Succinate Enhances HIF1A Protein Expression in METTL3-m6A-YTHDF1 Manner

**Figure 4:**
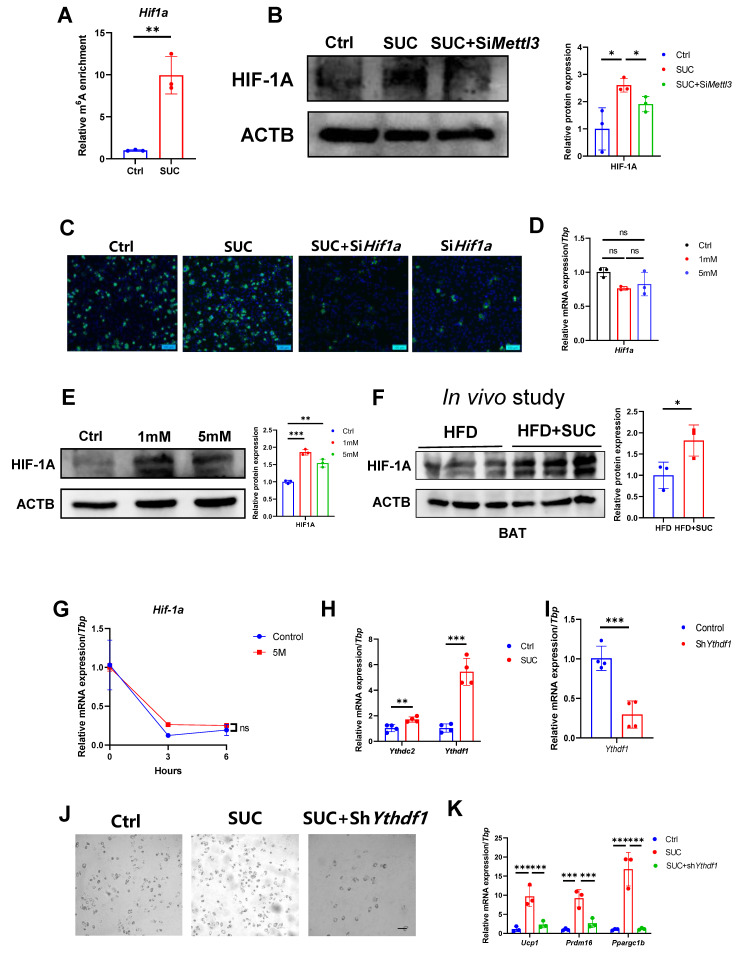
Succinate enhances HIF1A mRNA translation in METTL3-m^6^A-YTHDF1 manner. (**A**) The m^6^A levels on *Hif1a* mRNA in Ctrl or succinate-treated C3H10T1/2 cells (*n* = 3). (**B**) Western blot analysis of HIF1A protein in control, succinate-treated, and *Mettl3*-silenced groups of C3H10T1/2 cells. (**C**) Immunofluorescence of C3H10T1/2 cells in control, succinate-treated, or *Hif1a*-silenced groups. The green staining labeling for UCP1, blue staining for DAPI. Scale bar, 100 μm. (**D**) mRNA levels of *Hif1a* in primary brown adipocytes treated with 0, 1, and 5 mM succinate (*n* = 3). (**E**) Western blot analysis of HIF1A protein in primary brown adipocytes treated with 0, 1, and 5 mM succinate (**F**). Western blot analysis of HIF1A protein in HFD and HFD + SUC fed mice brown adipose tissue (*n* = 3) (**G**). Life-time of *Hif1a* mRNA in control, succinate-treated C3H10T1/2 cells (*n* = 3). (**H**) qPCR analysis of *Ythdc2* and *Ythdf1* in control or succinate treated C3H10T1/2 cells on day 6 of differentiation (*n* = 4). (**I**) qPCR analysis of *Ythdf1* mRNA level in control, *Ythdf1*-silenced groups C3H10T1/2 cells, (**J**) bright field of C3H10T1/2 cells in control, succinate treated, and *Ythdf1*- silenced groups. Scale bar, 50 μm. (**K**) qPCR analysis of *Ucp1*, *Prdm16*, and *Ppargc1a* expression in control, succinate treated, *Ythdf1*- silenced groups C3H10T1/2 cells on day 6 of differentiation (*n* = 3). The data were presented as the mean ± SD by Student’s *t*-test (**A**,**D**,**G**,**I**). * *p* < 0.05, ** *p* < 0.01, *** *p* < 0.001.

Previous studies showed that accumulated succinate may stabilize HIF-1A [23]. Meanwhile, our previous survey suggested that m^6^A modification increased 3T3-L1 cell thermogenic gene expression through HIF1A [18]. We hypothesized that succinate might regulate thermogenic gene expression through mediating HIF1A expression in a m^6^A-dependent manner. To elucidate whether succinate increases m^6^A in *Hif1a* mRNA, we used MeRIP-qPCR to measure m^6^A modification in *Hif1a* mRNA. As expected, succinate significantly increased m^6^A of *Hif1a* mRNA (Figure 4A). Meanwhile, loss-of-function studies showed that *Mettl3* was the crucial mediator, which is essential for increased HIF1A protein in succinate-treated C3H10T1/2 cells (Figure 4B). Furthermore, brown adipogenesis assays showed HIF1A deficiency attenuated brown adipogenesis (Figure 4C) and thermogenic gene expression. m^6^A modification plays several functions in mRNA metabolism, including translation, export, and mRNA stability. To elucidate the underlying mechanism m^6^A plays in HIF1A, we detected the *Hif1a* mRNA levels in succinate-treated cells. The results showed that succinate did not affect *Hif1a* mRNA expression of C3H10T1/2 cells (Figure 4D), whereas HIF1A protein expression was significantly elevated by succinate (Figure 4E). HIF1A protein expression of BAT was also elevated in HFD + SUC group mice (Figure 4F). We speculated that succinate might increase *Hif1a* mRNA stability; however, the life-time of *Hif1a* mRNA was not influenced by succinate (Figure 4G). Therefore, we hypothesized succinate augmented HIF1A expression through increasing translation. RNA-binding proteins have been unveiled that play vital roles in RNA metabolism [24,25,26]. Previous studies have demonstrated that m^6^A reader proteins YTHDF1 and YTHDC2 regulate translation. Meanwhile, we found succinate markedly increased *Ythdc2* and *Ythdf1* mRNA levels (Figure 4H). We performed knockdown of *Ythdf11* (Figure 4I), and the results showed that Sh*Ythdf1* markedly inhibited brown adipocyte biogenesis (Figure 4J) and thermogenic gene expression (Figure 4J). Taken together, these results indicate that succinate promotes HIF-1A protein expression in a m^6^A-YTHDF1-dependent manner.

### 2.5. Succinate Enhances Brown Adipose METTL3 Expression Through SDH

**Figure 5:**
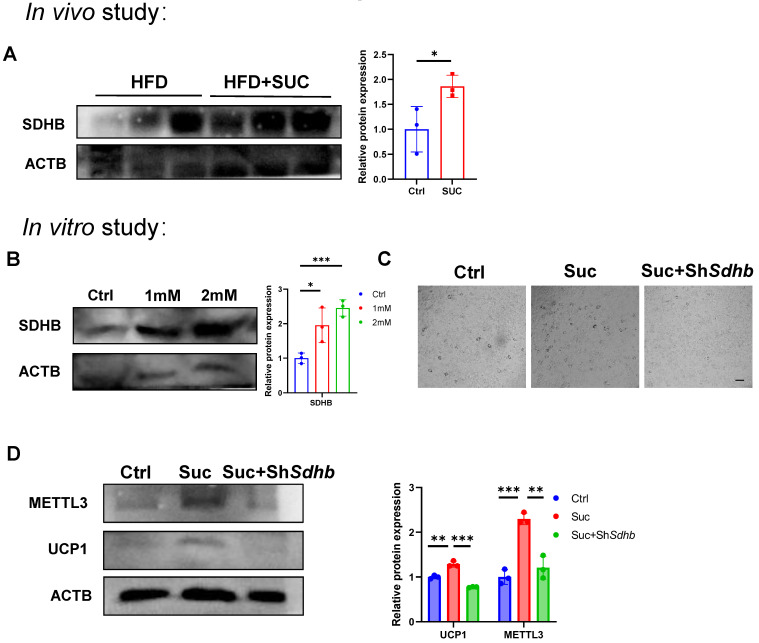
Succinate enhances brown adipose METTL3 expression through SDH. (**A**) Western blot analysis of SDHB in HFD and HFD + SUC group brown adipose tissue (*n* = 3). (**B**) Western blot analysis of SDHB in C3H10T1/2 cells treated with 0, 1, and 2 mM succinate. (**C**) bright field of C3H10T1/2 cells in control, succinate-treated, and *Sdhb*-silenced groups. Scale bar, 50 μm. (**D**) Western blot analysis of METTL3 and UCP1 in control, succinate-treated, and *Sdhb*-silenced groups. The data were presented as the mean ± SD by Student’s *t*-test (**A**,**B**,**D**). * *p* < 0.05, ** *p* < 0.01, *** *p* < 0.001.

Succinate dehydrogenase (SDH) is a key metabolic enzyme in the tricarboxylic acid cycle and a component in the electron transport chain (ETC). Previous studies have shown that succinate promotes SDH expression in the mitochondrial membrane and catalyzes succinate to fumarate [6]. Meanwhile, intracellular succinate may regulate m^6^A-related protein expression [19]. In this study, we found that succinate significantly elevated SDHB protein expression in brown adipose tissue (Figure 5A). And our results show that SDHB protein expression in C3H10T1/2 cells was markedly increased following succinate treatment (Figure 5B). To further verify the *Sdhb* function, we conducted a loss-function assay, and a western blot showed sh*Sdhb* significantly decreased SDHB protein expression in C3H10T1/2 cells (Figure S4A). The results suggested that *Sdhb* deficiency inhibited brown adipogenesis, which was promoted by succinate (Figure 5C). Furthermore, *Sdhb* deficiency hampered METTL3 and UCP1 expression (Figure 5D), which was increased by succinate treatment. Altogether, *Sdhb* is an upstream regulator of METTL3, and succinate enhanced brown adipocyte METTL3 expression through *Sdhb*.

## 3. Discussion

Here, we showed that succinate supplementation effectively ameliorated obesity and glucose homeostasis through promoting brown adipose tissue thermogenesis. Of note, in our study, we found that succinate could enter the circulatory system, ultimately promoting brown adipose tissue thermogenesis through an m^6^A-associated manner.

Succinate, the key tricarboxylic acid cycle metabolite, has been revealed to play crucial roles in energy metabolism, and acts as a signal molecule that regulated cytokines secretation [22,27]. Research suggested that individuals with higher circulating succinate levels have greater visceral adipose tissue (VAT) mass and triglyceride levels, which are potential biomarkers of high cardiovascular disease risk [28,29]. However, several surveys showed succinate accumulation in brown adipocytes promotes brown adipose tissue thermogenesis, thereby combating obesity [6,22]. In this study, we found that dietary succinate can infiltrate the circulatory system directly, leading to elevated plasma succinate levels. Meanwhile, we verified that the succinate concentration in the plasma of HFD + SUC-fed mice promotes brown adipogenesis in vitro, which prevents HFD-induced obesity in mice. Traditionally, serving as a tricarboxylic acid metabolite, succinate was considered to regulate superoxide production via the mitochondrial respiratory chain. Superoxide/H_2_O_2_, driven by succinate, oxidized protein cysteine thiols, and then the modified protein boosted thermogenesis. In the process, succinate dehydrogenase plays a crucial function that transfers succinate to fumarate, leading to ROS production. Therefore, we speculated that an abnormal mitochondrial respiratory chain may be unable to consume intracellular succinate, contributing to excessive succinate entering the circulatory system. The abnormal mitochondrial respiratory chain reduced futile cycle, which led to higher fat mass [30]. Increased succinate caused by exogenous addition may augment energy in brown adipocytes. In these studies, we show that 1.5% succinate in drinking water elevates succinate levels in the circulatory system, and the increased succinate promotes brown adipose thermogenesis.

Mechanistically, previous studies indicate succinate accumulation may involve PTMs, including lysine succinylation [29]. SDH plays an essential role in protein modification, and it regulates the metabolic reprogramming process via ROS production [31]. As the substrate of SDH, succinate accumulated when SDH was mutated [26,27].

Previous studies have shown that elevated succinate in MSCs inhibits fat mass and FTO, thereby enhancing m^6^A methylation [19]. And our studies previously showed that BCAAs supplementation alleviates high-fat diet (HFD)-induced obesity through inhibiting FTO expression of adipocytes [32]. METTL3 has been identified to regulate bone marrow mesenchymal stem cell (BMSCs) differentiation via the Janus kinase 1 (JAK1) signaling pathway [27]. Meanwhile, Xie et al. show that METTL3 is a vital regulator that controls BAT postnatal development and energy homeostasis [17]. However, there was no evidence that succinate directly regulates METTL3 expression, thereby influencing thermogenesis in an m^6^A-dependent manner. In this study, we validated that the METTL3 protein increased in a succinate dose-dependent manner.

HIF1A, the key protein, plays crucial roles in signal transduction under hypoxic conditions [33,34]. And HIF1A has been demonstrated to facilitate brown adipose thermogenesis, which is the transcription factor of thermogenic genes [18,35].

Succinate triggers ROS production through succinate dehydrogenase and stabilizes HIF-1A protein via posttranslational modification [36]. And our previous studies show that m^6^A participates in *Hif1a* translation [18]. The key question is whether succinate influences HIF1A expression in a m^6^A-dependent manner. In this study, we conducted MeRIP-qPCR and unveiled that succinate supplement elevates the m^6^A level of *Hif1a* mRNA. More importantly, we found that *Ythdf1* expression in C3H10T1/2 cells is markedly elevated by succinate treatment. Using a loss-of-function assay, we found that *Ythdf1* is essential for succinate-induced brown adipogenesis and regulates HIF1A protein expression.

Altogether, we show that succinate supplementation protects against HFD-induced mice obesity by promoting energy expenditure in an SDH-METTL3-m^6^A-HIF1A manner. And the effects of the accompanying increase in reactive oxygen species with succinate supplementation need further research.

## 4. Materials and Methods

### 4.1. Animals

20 seven-week-old male C57BL/6J mice were purchased from Vital River (Beijing, China). Mice were housed in individual cages under a 12 h light-dark cycle, 23 ± 2 °C room temperature, and 40–60% humidity, with ad libitum access to food. After acclimation for two weeks, the mice were randomly divided into two groups (*n* = 10/group), and fed either high-fat diets (HFD, 60 kcal% high-fat diets; research diet 12492) or high-fat diets with 1.5% (*w*/*w*) succinate (Sigma 14160-100G, St. Louis, MO, USA) in drinking water (HFD + SUC). 1.5% (*w*/*w*) succinate was dissolved in sterile water. Body weights were recorded once a week. Before being killed, mice were anesthetized, then epididymal white adipose tissue (eWAT), subcutaneous inguinal white adipose tissue (iWAT), brown adipose (BAT), and liver were immediately frozen in liquid nitrogen and stored at −80 °C. For plasma collection, the blood samples were centrifuged at 3000 rpm at 4 °C for 15 min, and the serum was collected and stored at −80 °C. Food intake was measured once a week. All animal experiments were approved by the Committee on Animal Care and Use and the Committee on the Ethics of Animal Experiments of Zhejiang University (ZJU20240145).

### 4.2. Glucose Tolerance Test (GTT) and Insulin Tolerance Test (ITT)

After 16 h of fasting, mice were subjected to a glucose tolerance test (GTT). In Brief, 16 h-fasted mice were injected with D-glucose (1.5 g/kg body weight, Sigma-Aldrich, St. Louis, MO, USA). For the insulin tolerance test, mice were injected with insulin (0.75 U/kg body weight, Sigma-Aldrich, St. Louis, MO, USA) after 4 h of fasting. For both tests, glucose levels were measured by the glucometer (Yuyue Electronics, Nanjing, China) at baseline and at the indicated time points (15, 30, 60, 90, 120 min) after intraperitoneal injection.

### 4.3. Cold Exposure Experiment

For the acute cold experiment, mice were individually housed and placed in a fridge (4 °C) for 4 h with free access to food and water. Core body temperature was measured with a rectal probe (TH212) at the indicated time points.

### 4.4. Serum Succinate Detection

Mice serum succinate was detected according to the manufacturer’s instructions of the succinate assay kit (Sigma-Aldrich, MAK335-1KT).

### 4.5. Cell Culture and Brown Adipocyte Differentiation

Isolation of primary brown preadipocytes from BAT and induction of preadipocyte differentiation were performed as previously reported. Briefly, the BAT was excised from 6-week-old C57BL/6 mice, minced, and then digested with 2 mg/mL type II collagenase (Thermo Fisher, Waltham, MA, USA) at 37 °C for 45 min with shaking (180 rpm). The mice were fed a normal Diet. Digests were then filtered through 70- and 40-μm mesh into 15 mL Falcon tubes. Visible fat floating as a layer was carefully aspirated. The pellets were seeded in DMEM supplemented 10% fetal bovine serum (FBS; Gibco, Grand Island, NY, USA) and 1% penicillin-streptomycin at 37 °C in a humidified incubator with a 5% CO_2_. For adipocyte differentiation, cells were induced in medium containing 0.5 mM IBMX, 1 µM dexamethasone, 5 µg/mL insulin, 1 nM T3, 125 nM indomethacin, and 10% FBS for 2 days. Cells were transferred to maintenance medium consisting of 10%FBS, 5 µg/mL insulin, and 1 nM T3 for another 5 days. C3H10T1/2 cells were purchased from the National Collection of Authenticated Cell Cultures. For adipocyte differentiation, C3H10T1/2 cells were induced in medium containing 0.5 mM IBMX, 1 µM dexamethasone, 5 µg/mL insulin, 1 nM T3, 125 nM indomethacin, and 10% FBS for 2 days. Then, the cells were transferred to maintenance medium consisting of 10%FBS, 5 µg/mL insulin, and 1 nM T3 for another 5 days. Succinate treatment is as follows: after confluence of primary brown preadipocytes and C3H10T1/2 cells, the MDI and the maintenance medium are supplemented with 0, 1, 2, 5 and 10 mM succinate.

### 4.6. Cell Transfection, Plasmids, and RNA Knockdown

The siRNA and plasmid transfection were performed with Lipofectamine 3000 (Thermo Fisher, L3000001), according to the manufacturer’s instructions. All siRNAs and plasmids were synthesized by Genepharma (Shanghai, China). The sequence of negative control siRNA is as follows (5′ to 3′): 5′-UUCUCCGAACGUGUCACGUTT-3′. Mouse *Mettl3* siRNA targets 5′-CUGCAAGUAUGUUCACUAUGATT-3′. Mouse si*Hif*1a siRNA targets 5′-GACACAGCCUCGAUAUGAATT-3′. Mouse si*Ythdc2* siRNA targets 5′-CCUGUUAGAUGAUUGCUUUTT-3′. The sh*Sdhb* plasmid was generated by cloning the full-length ORF of the mouse *Sdhb* gene into the LV3 vector (H1). The sh*Ythdf1* plasmid was generated by cloning the full-length ORF of the mouse *Ythdf1* gene into the LV3 vector (H1). The OE-*Mettl3* plasmid was generated by cloning the *Mettl3* gene (NM_019721.2) into a pcDNA3.1 vector.

### 4.7. 3D Cell Culture

Primary mouse eWAT preadipocytes were seeded into 10 cm^2^ cell culture dishes containing culture medium (DMEM/F12, supplemented with 15% FBS and 1% penicillin–streptomycin). Afterward, the cells were passaged, trypsinized, and seeded into a 96-well round-bottom ultra-low attachment plate at 8000 cells per well. After 2 days, the culture medium was replaced with MDI medium containing 1 μmol/L dexamethasone (Sigma-Aldrich, St. Louis, MO, USA), 500 μmol/L IBMX (Sigma-Aldrich, St. Louis, MO, USA), 1 μg/mL insulin (Sigma-Aldrich, St. Louis, MO, USA), and 1 μM rosiglitazone. After 3 days, the medium was replaced with differentiation medium containing 5 μg/mL insulin.

### 4.8. Quantitative Real-Time PCR (RT-qPCR)

Total RNA from brown adipose tissue, brown adipocytes, and 3T3-L1 cells was extracted and purified using RNAiso Plus (Takara, Kusatsu, Japan), and the RNA concentration was analyzed by Nanodrop 2000 (Thermo Fisher, Waltham, MA, USA). Then the RNA was reverse transcribed into cDNA by M-MLV reverse transcriptase (ABclonal RK20429, Wuhan, China). qPCR was performed using SYBR Green PCR Master Mix (ABclonal, RK21203, Wuhan, China) with the ABI Step-One PlusTM Real-Time PCR System (Applied Biosystems, Foster City, CA, USA) or CFX touch 96 (Bio-Rad, Hercules, CA, USA). The data analysis followed the 2^−ΔΔCt^ method and was calculated using *Tbp* as the internal control. The primers used for RT-qPCR are listed in Table S1.

### 4.9. Protein Extraction and Western Blot

Firstly, tissues and cells were washed twice with cold PBS, and lysed in cold RIPA buffer (Beyotime Biotechnology, Shanghai, China, P0013B) with protease (Beyotime Biotechnology, P1045) and phosphatase inhibitor cocktail (Proteintech, Wuhan, China, PR20015) for 30 min. After using the BCA protein assay kit (Yeasen, Shanghai, China, 20201ES76) to detect the protein concentration. 10 µg total protein sample was electrophoresed in SDS–polyacrylamide gels and transferred to PVDF membrane (Millipore, Burlington, MA, USA, IPVH00010) After blocking the 5% non-fat milk at room temperature for 1 h, the membrane was incubated with a 1:1000 dilution of corresponding primary antibodies overnight at 4 °C. After three washes or 30 min, the membrane was incubated with a 1:10,000 dilution of anti-rabbit HRP-conjugated secondary antibodies (Proteintech, SA00001-2) at room temperature for 1 h. After two washes for 30 min, protein bands were visualized by ECL (Biosharp, Hefei, China, bl520A). The antibodies in the experiment are listed in Table S2.

### 4.10. mRNA m6A Level Detection

Dot blots were carried out to detect the m^6^A level in the mRNA of brown adipose tissue and cells. The m^6^A dot blot assay was conducted as previously described. In brief, the mRNA was purified from total RNA using the Dynabeads mRNA DIRECT kit (Invitrogen, Waltham, MA, USA, 61006) according to the manufacturer’s protocols. The mRNA was denatured at 65 °C for 5 min, then chilled on ice. Then, 200 ng of mRNA was added to an Amersham Hybond-N^+^ membrane (GE Healthcare, Chicago, IL, USA) and cross-linked twice at 254 nm using the apparatus (Pharmacia Biotech, Uppsala, Sweden, UVC500-230V). After UV crosslinking, the membrane was then washed with 1× PBST buffer, blocked with 5% non-fat milk in PBST, and incubated with anti-m^6^A antibody (202003, 1:800; Synaptic Systems, Göttingen, Germany) for 4 °C. After incubating with HRP-conjugated anti-rabbit secondary antibody (Poteintech, SA00001-2), followed by imaging using a ChemiScope 6000 Imaging system (CLINX, Shanghai, China). Finally, the membrane was stained with 0.02% methylene blue as a control.

### 4.11. Methylated RNA Immunoprecipitation-qPCR (MeRIP-qPCR)

MeRIP-qPCR was conducted according to previous reports. Briefly, extracted RNA was transferred into 1.5 mL Eppendorf tubes, then fragmented using a ultrasonic homogenizer (Bioruptor, Seraing, Belgium). The sample was incubated with m^6^A antibody (Synaptic Systems, Göttingen, Germany), coated protein A and protein B beads (Thermo Fisher, Waltham, MA, USA) at 4 °C for 4–5 h in IP buffer (150 mM NaCl,10 mM Tris-HCl, 0.1% NP40 in nuclease-free H_2_O), and a small part of the fragmented RNA was used as input RNA. The sample was placed into a magnet for 1 min. Then the beads were washed with IP buffer twice, low-salt buffer twice, and high-salt buffer twice. Each time, rotate the tube at 4 °C for 5 min. The beads were suspended in 200 μL of RNAiso Plus reagent and extracted with the same method as used for RNA extraction. The input and IP samples were used for reverse transcription and RT-qPCR. The primer sequences are presented in Table S1.

### 4.12. mRNA Decay Analysis

RNA stability analysis was performed as described previously. Briefly, control and succinate-treated cells were treated with 5 µg/mL actinomycin D (Sigma-Aldrich, A9415) to inhibit mRNA transcription. Samples were collected at 0, 3, and 6 h. The RNA was extracted for reverse transcription, and mRNA levels of the target gene were analyzed by qPCR.

### 4.13. Triglyceride Assay

Triglyceride assay in serum was performed according to the manufacturer’s instructions of the triglyceride assay kit (Nanjing Jiancheng Bioengineering Institute, A110-1-1).

### 4.14. Blood Glucose Assay

For the blood glucose assay, the mice were fasted overnight, and their tails were cleaned by 70% ethanol. Then, the blood glucose level was detected in tail blood using a glucometer with glucose test strips (Yuyue Electronics, Nanjing, China).

### 4.15. Immunofluorescence

Cells were rinsed twice with PBSS, then incubated in 4% paraformaldehyde (PFA) for 20 min and permeabilized with 0.2% Triton X-100 for 10 min. Subsequently, the cells were blocked with 5% goat serum for 1 h at room temperature. Afterward, cells were incubated with primary antibodies (1:200, UCP1) at 4 °C overnight. Subsequently, goat anti-rabbit Alexa Fluor 488 (Abcam, Cambridge, UK, 1:200) antibodies were incubated at room temperature for 1 h. Then the cells were washed three times and stained with Nile red (1:8000) for 30 min. Finally, the cells were washed 3 times with PBS and stained with DAPI for 10 min. Images were acquired using IX-85 (Olympus, Tokyo, Japa).

### 4.16. Histological and Image Analysis

All tissues were fixed in 4% paraformaldehyde for 24 h at 4 °C, then dehydrated and embedded in paraffin. Subsequently, Slides (5 μm) were cut with a Leica RM 2235 (LEICA, Wetzlar, Germany), then the sections were prepared and stained with hematoxylin and eosin. Images were acquired at 20× magnification using DM3000 (LEICA, Wetzlar, Germany), and the adipocyte sizes were analyzed with ImageJ (1.48q, Bethesda, MD, USA).

### 4.17. Statistical Analysis

All data were presented as the mean ± SD. Significance between the two groups was tested by using Student’s *t*-test using GraphPad Prism 9. One-way ANOVA followed by Tukey’s test was used for multiple group comparisons; significance was established at *p* < 0.05.

## 5. Conclusions

In conclusion, we found that succinate supplementation alleviated HFD-induced obesity through brown adipose tissue thermogenesis in SDH-METTL3-m^6^A-HIF1A manner in mice.

Dietary succinate increases SDH expression in mice brown adipocytes, which is the upstream factor of METTL3. And augmenting METTL3 promotes brown adipocyte thermogenesis through elevating the m^6^A level of *Hif1a*, which increases HIF1A expression, contributing to facilitating thermogenic gene expression. Our study demonstrated that succinate prevents obesity and promotes brown adipocyte thermogenesis in an m^6^A-orchestrated manner and provides crucial evidence that dietary succinate enters the circulatory system and directly boosts brown adipocyte thermogenesis.

## Data Availability

All materials and data supporting this study are available from the author (yaojunluo@zju.edu.cn) upon reasonable request.

## Supplementary Materials

The following supporting information can be downloaded at: https://www.mdpi.com/article/10.3390/ijms27125348/s1.
