# Supplementary material for: Succinate Prevents Mice Obesity by Enhancing Brown Adipocyte Thermogenesis via the SDH-METTL3-HIF1A Pathway
Source: Int J Mol Sci. 2026 Jun 13;27(12):5348. doi: 10.3390/ijms27125348 (PMC13299057; doi:10.3390/ijms27125348)
Supplement: Supplementary file 1 [file ijms-27-05348-s001.zip › Figures legends.pdf]

Figure S1. (A). A Food intake of mice in HFD and HFD+SUC group (B) Water intake of mice HFD and HFD+SUC group. Two-way ANOVA was followed by
Bonferroni's test was used for repeated measurement of two groups (A, B). *p < 0.05.

Figure S2. (A) Succinate level of mice serum in HFD and HFD+SUC group (n=5). (B) qPCR analysis of *Pparg*, *Cebpa*, and *Prdm16* mRNA level in iWAT of HFD and HFD+SUC group mice (n=3). (C) qPCR analysis of *Pparg*, *Cebpa,* and *Hsl* mRNA levels in iWAT of HFD and HFD+SUC group mice (n=3). (D) Representative bright field images of iWAT SVF cells treated with 0, 1, 5, and 10 mM succinate.Scale bar,100 μm. (E) Representative immunofluorescence of 3D cultured eWAT preadipocytes treated with 0, 1, and 5 mM succinate. Scale bar,50 μm. (F) Immunofluorescence of C3H10T1/2 cells treated with 0, 1, 2, 5, and 10 mM succinate . Scale bar, 50μm. (G) qPCR analysis of *Ucp1* mRNA level in C3H10T1/2 cells (n=3). The data were presented as the mean ±SD by Student's t-test (A, B, C, G). *p < 0.05, **P < 0.01, ***P < 0.001.

Figure S3. (A) Dot blot analysis for total m6A level in C3H10T1/2 cells of control, succinate-treated, and Mettl3-silenced groups. (B) Representative bright field images of control and *Mettl3*-overexpressing C3H10T1/2 cells. Scale bar,100 μm. (C) qPCR analysis of Ucp1 mRNA level in control, vector, and Mettl3-overexpressing C3H10T1/2 cells (n=3). (D) Western blot analysis of METTL3 and UCP1 protein expression in vector and *Mettl3*-overexpressing C3H10T1/2 cells. The data were presented as the mean ±SD by Student's t-test (C, D). *p < 0.05, ***P < 0.001.

Figure S4. (A) Western blot analysis of SDHB protein expression in control and *Sdhb*-silenced C3H10T1/2 cells. The data were presented as the mean ±SD by Student's t-test. *p < 0.05.
