# Supplementary figures and images for: Succinate Prevents Mice Obesity by Enhancing Brown Adipocyte Thermogenesis via the SDH-METTL3-HIF1A Pathway
Source: Int J Mol Sci. 2026 Jun 13;27(12):5348. doi: 10.3390/ijms27125348 (PMC13299057; doi:10.3390/ijms27125348)

# Supplement Figure S1

**A**

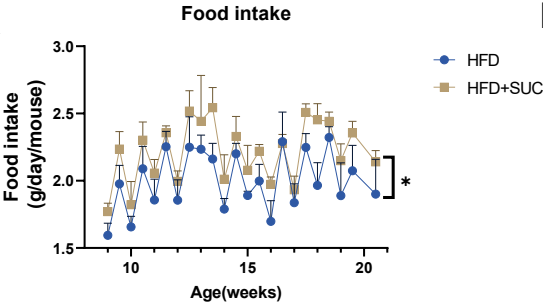

**B**

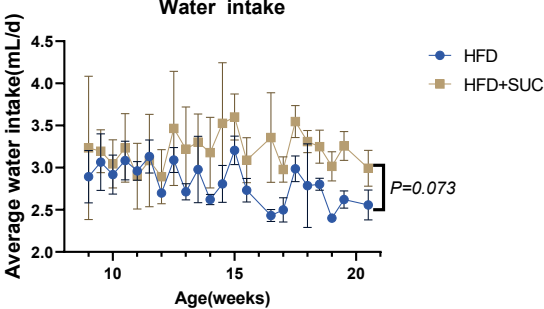

# Supplement Figure S2

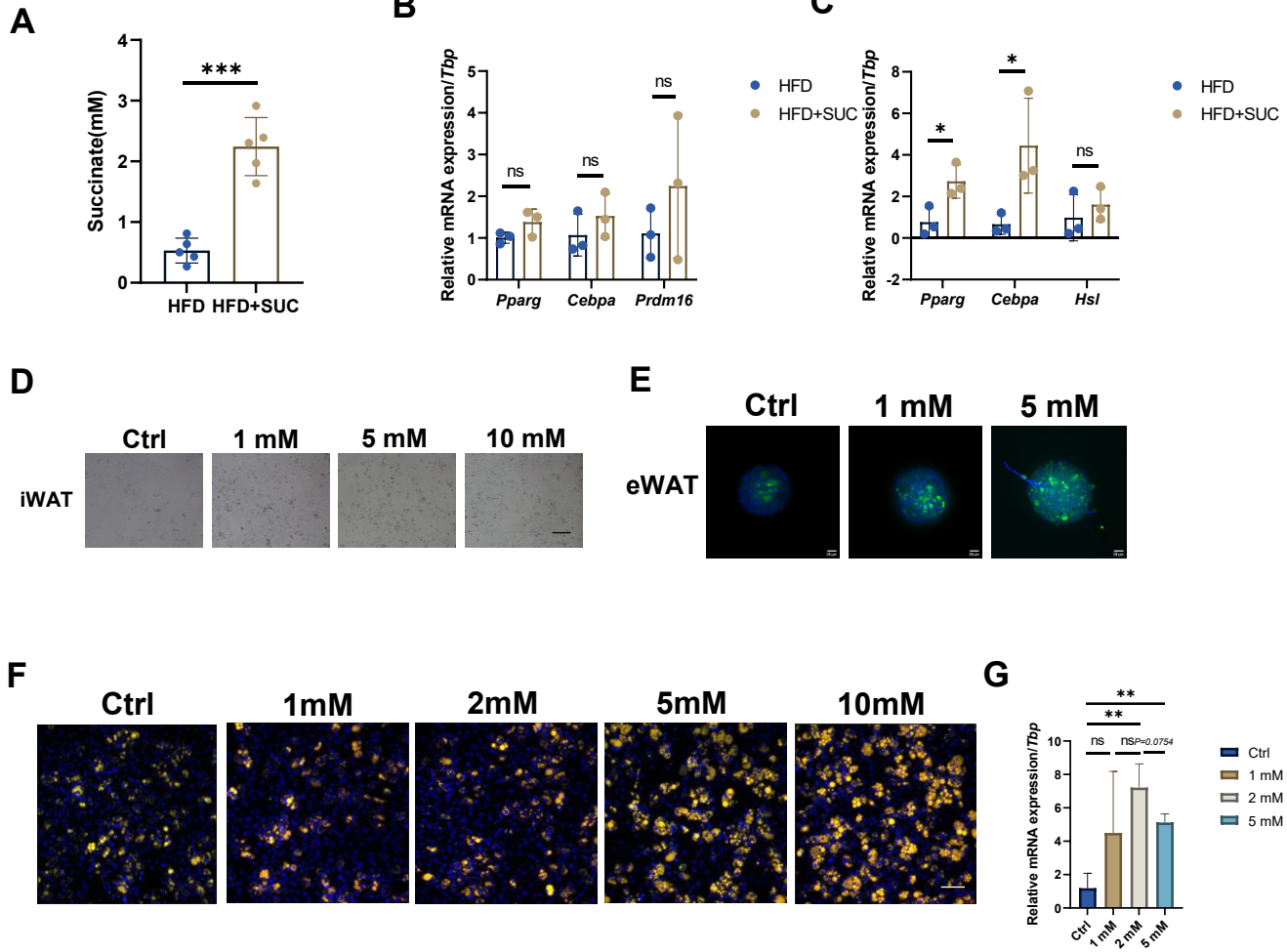

# Supplement Figure S3

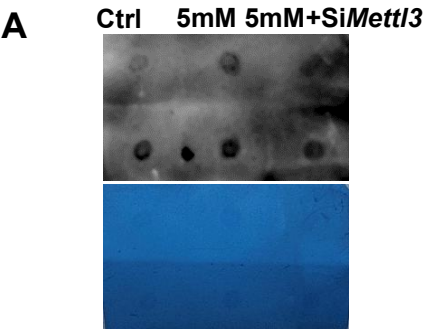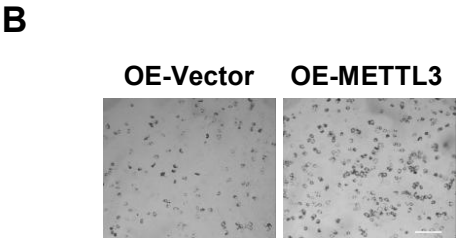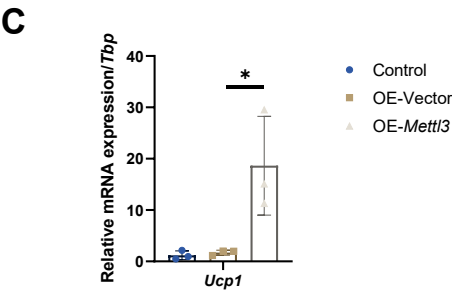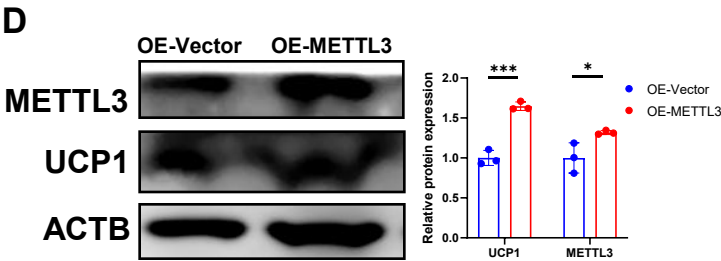

# Supplement Figure S4

A

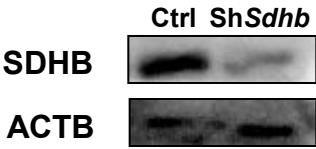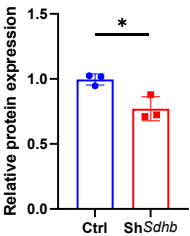

Supplement: Supplementary file 1 [file ijms-27-05348-s001.zip › Supplementary Figures S1-S4.pdf]
